# Associations Between Nutritional Status, Sociodemographic Characteristics, and Health-Related Variables and Health-Related Quality of Life Among Chinese Elderly Patients: A Multicenter Prospective Study

**DOI:** 10.3389/fnut.2020.583161

**Published:** 2020-10-16

**Authors:** Hongpeng Liu, Jing Jiao, Chen Zhu, Minglei Zhu, Xianxiu Wen, Jingfen Jin, Hui Wang, Dongmei Lv, Shengxiu Zhao, Xinjuan Wu, Tao Xu

**Affiliations:** ^1^Department of Nursing, Peking Union Medical College Hospital, Chinese Academy of Medical Sciences & Peking Union Medical College, Beijing, China; ^2^Department of Geriatrics, Peking Union Medical College, Peking Union Medical College Hospital, Chinese Academy of Medical Sciences, Beijing, China; ^3^Department of Nursing, Sichuan Provincial People's Hospital, Chengdu, China; ^4^Department of Nursing, The Second Affiliated Hospital Zhejiang University School of Medicine, Hangzhou, China; ^5^Department of Nursing, Tongji Medical College, Tongji Hospital, Huazhong University of Science and Technology, Wuhan, China; ^6^Department of Nursing, The Second Affiliated Hospital of Haerbin Medical University, Haerbin, China; ^7^Department of Nursing, Qinghai Provincial People's Hospital, Xining, China; ^8^Department of Epidemiology and Statistics, Institute of Basic Medical Sciences, Chinese Academy of Medical Sciences & School of Basic Medicine, Peking Union Medical College, Beijing, China

**Keywords:** aging, malnutrition, depression, frailty, health-related quality of life, China

## Abstract

**Background:** Studies that explore the nutritional status, sociodemographic factors, mental health variables, and physical health variables that affect the health-related quality of life (HRQoL) of elderly patients are scarce in China.

**Objective:** This study aimed to examine the association between health-related quality of life (HRQoL) and nutritional status, sociodemographic characteristics, and health-related variables among Chinese elderly patients.

**Materials and Methods:** Participants were recruited from six tertiary-level hospitals in six provinces or municipalities/cities throughout China from October 2018 to February 2019: a total of 9,996 participants aged 65 years and older were enrolled. The nutritional status and HRQoL were measured using the Mini Nutritional Assessment—Short Form (MNA-SF) and the EuroQoL Five-Dimension Visual Analog Scale, respectively. BMI was taken using standard measurement protocols. Sociodemographic characteristics included age, sex, education, marital status, ethnicity, smoking, alcohol drinking, and current residence. Mental and physical health variables such as frailty and depression were assessed using validated tested instruments. Multiple linear regression analysis was used to analyze whether the nutritional status, sociodemographic characteristics, and health-related variables were associated with HRQoL.

**Results:** According to the MNA-SF scores at the 30- and 90-day follow-up, 9.7% and 9.1% of participants were malnourished, respectively. Higher MNA-SF scores were related to higher HRQoL scores in older patients (regression coefficient; 95% confidence interval) both at the 30-day (0.660; 0.499–0.821) and 90-day (0.622; 0.434–0.809) follow-up. However, there were no significant associations between the body mass index values and HRQoL. Sociodemographic characteristics (such as age, smoking, and current residence), physical health variables (frailty, urinary function, defecation function, sleeping condition, and falling accidents in the past 12 months), and mental health variables (depression) were the main factors influencing HRQoL in this group.

**Conclusion:** There are several factors associated with HRQoL among the population derived from this investigation of a representative sample of the Chinese hospitalized elderly population in tertiary hospitals. These findings could have major importance for the planning of “active aging” policies and programs.

**Trial Registration:** Chinese Clinical Trial Registry, ChiCTR1800017682, registered August 9, 2018.

## Introduction

The aging population is growing rapidly worldwide and looks set to continue to increase even further in the future (1, 2). For example, by 2050, one-fifth of the world's population will be aged 60 years or older, and the proportion of the older population is expected to double from about 11% in 2000 to 22% in 2050 (3). In 2018, the number of Chinese older adults approached 241 million, accounting for 17.2% of the total population, and this figure is expected to approach 480 million by 2050 (1, 4).

Aging in humans may be accompanied by physiological and pathological changes relevant to nutrition, social, psychological, and physical factors, such as weight loss, impaired appetite, depression, and functional disability (5–9). There is growing evidence that malnutrition is a common health problem among older adults in hospital, community, and nursing home settings (5, 10, 11). The burden of malnutrition among the Chinese elderly is serious, and in 2017, for example, malnutrition increased hospital costs by RMB 214 (14% increase) per older adult (12). Also, poor mental health and physical functioning contributes to a progressive decline in health, hindering recovery from illness and leading to increased utilization of health care services and premature institutionalization (6, 13, 14).

Health-related quality of life (HRQoL) is a subjective, multidimensional measure reflecting functional status and emotional and social well-being as well as general health (8, 15). HRQoL has become a main goal in the promotion and development of health among older adults (8, 16, 17). Deterioration of physical functioning can result in difficulties with daily activities including cooking and eating, which may affect nutritional status (2, 4). Malnutrition and declining functional status are two critical factors related to loss of independence with aging (18). Therefore, low HRQoL in older adults can reflect potential health problems contributing to functional disability and dependence as well as a risk of malnutrition (19, 20).

Previous studies have shown a direct association between poor nutritional status and worse HRQoL in some groups, such as adults aged 80 years and older (16). Most previous studies have used differing nutritional screening tools, such as the Mini Nutritional Assessment (MNA) and Malnutrition Universal Screening Tool (MUST) (8, 17), as well as estimated nutritional status and HRQoL in older adults using data from only a single hospital or a smaller sample size (8, 16, 17, 21). Studies conducted in China have explored frailty, depression, and the association between HRQoL and functional abilities among the elderly (22–25), but only a few studies have focused on the association between nutrition and HRQoL among elderly patients. Therefore, studies that explore the sociodemographic factors, nutritional status, mental health variables, and physical health variables that affect the HRQoL of the elderly are scarce in China. To address this issue, we conducted a study designed to examine the association between nutritional status, sociodemographic characteristics, health-related variables, and HRQoL in Chinese elderly patients aged 65 years and above based on a large-scale prospective national survey.

## Materials and Methods

### Study Design and Participants

The participants were derived from a large-scale cohort study in a representative sample of the Chinese hospitalized elderly population in tertiary hospitals, which is an ongoing survey of the physiological and psychological conditions in elderly patients nationwide (Chinese Clinical Trial Registry number ChiCTR1800017682). The baseline data collected from the period from October 2018 to February 2019 represent the baseline survey data used in this study.

The target population is all older inpatients in tertiary hospitals. Eligible participants were recruited using a two-stage cluster sampling method to ensure the representativeness of the study sample. In the first stage, five provinces and one municipality/city in China (southwest: Sichuan Province; northeast: Heilongjiang Province; south central: Hubei Province; northern: Beijing municipality/city; northwest: Qinghai Province; eastern: Zhejiang Province) were selected. A simple random sampling method was used in this stage. In the second stage, one tertiary hospital was selected in each province or municipality/city: The Peking Union Medical College Hospital (PUMCH), where the author works, as a form of convenience sampling. In addition to this hospital, five other hospitals were selected through simple random sampling. All eligible elderly patients from the surgical, internal medicine, neurology, and orthopedics departments and the intensive care unit (ICU) of the selected hospitals were continuously enrolled.

A sample size of 7,299 can produce a two-sided 95% confidence interval with a tolerance error equal to 0.005 when the predicted prevalence rate is 5% (26). Considering the potential non-response and loss to follow, 10,000 participants will be recruited in this study. The inclusion criteria were as follows: aged 65 years and older; signed the consent form; understood the aims of the study; and with sufficient mental ability to answer the interview questionnaire.

### Measurement Instruments

The Mini Nutritional Assessment—Short Form (MNA-SF) is a six-item scale that assesses nutritional risk. Assessment is a validated test with sensitivity and specificity for the diagnosis of malnutrition, especially for the elderly (5). Responses yield a score from 0 to 14 points. The participants were then categorized into normal nutritional status (12–14 points), at risk for malnutrition (8–11 points), or malnourished (0–7 points) (6). The MNA-SF has been validated in the Chinese population and has excellent test characteristics (27).

Weight (in kilograms), height (in centimeters), and body mass index (BMI) were recorded. The participants were weighed in light clothing with the footwear removed. The weight of the participants was measured to the nearest 0.1 kg using digital electronic chair scales and the height to the nearest 1 mm using a portable stadiometer. BMI was calculated by dividing the body weight by the height squared (in kilograms per square meter) (28) and was used to classify participants into groups of emaciation (<18.5 kg/m^2^), normal (18.5–23.9 kg/m^2^), overweight (24–27.9 kg/m^2^), and obesity (≥28 kg/m^2^) according to the Chinese guidelines for the prevention and control of adult overweight and obesity (29) and the Chinese criteria by the Working Group on Obesity in China (WGOC) (30).

HRQoL was measured using the EuroQoL Five-Dimension Visual Analog Scale (EQ5DVAS), in which the participants were asked to rate their overall health status. Possible scores range from 0 to 100, in which 0 represents the worst imaginable health state and 100 the best imaginable health state (8, 13, 16, 31). This scale has been validated among the Chinese population (32, 33).

### Data Collection and Quality Control

The data on nutritional status, sociodemographic characteristics, and health-related variables were collected by 589 well-trained and certified registered nurses who conducted face-to-face questionnaire interviews, health assessment, and physical examinations and reviewed clinical records. To ensure data quality, firstly, the research group developed the project survey manual, operation manual, and training manual. Secondly, a database was built using an electronic data collection (EDC) system to guarantee the accuracy and integrity of the data. Also, to ensure accurate data collection, all nurses received systematic training on completing the case report form (CRF) before they recorded the patients' information daily on the web-based online CRF; they were proficient in the investigation process, the method of using the EDC system, and the application of the depression assessment, frailty assessment, and other health assessment scales. Data on HRQoL were collected at both 30 and 90 days in a telephone follow-up.

### Definition of Covariates

We developed a multiple linear regression model including factors potentially associated with HRQoL: age, sex, education, marital status, ethnicity, smoking, alcohol consumption, falling accidents in the past 12 months, sleeping condition, urinary function, defecation function, frailty, depression, and current residence. Sleeping condition, urinary function, and defecation function were dichotomized as normal function or dysfunction. Frailty was assessed using the Frailty Scale (34), with a higher total score indicating a more severely frail condition, which represents frail (3–5), pre-frail (1, 2), and robust (0). The FRAIL scale has been validated for use in older Chinese population (22). Depression assessment was based on the 15-item Geriatric Depression Scale 15 (GDS-15) (35). The total GDS score was the sum of the responses of the 15 depression questions (range, 0–15). If the total GDS score is above 5, the patient was considered as depressed, with a larger GDS-15 score denoting more severe depression. The GDS-15 has been validated for use in Chinese elderly (36).

### Statistical Analysis

A descriptive analysis of the participants' characteristics was performed using proportions and measures of central tendency and dispersion, in accordance with the nature of the variables. Normality probability plots showed that the EQ5DVAS scores were normally distributed. Multiple linear regression analysis was used to predict EQ5DVAS scores according to the sociodemographic characteristics, health-related variables, and nutritional status. Variables that were not multicollinear were entered into the multiple linear regression model. Regression coefficients and 95% confidence intervals (CIs) were used to assess the strength of the relationships. All statistical analyses were conducted using SAS 9.4 software (SAS Institute Inc., Cary, NC, USA). Statistical significance was accepted at the level of 0.05.

### Ethics

This study received approval from the ethics committee of the Peking Union Medical College Hospital (S-K540). All participants signed a form consenting to participate in this study, which adhered to the principles of the Declaration of Helsinki. The participants and their families were provided detailed information about the aim of the study, the terms of participation in the study, the rights of the participants, and questions to be asked. The interview language used is standard Mandarin/Putonghua. If the participants had specific conditions, such as cognitive decline, the nurse interviewed a legal guardian or representative who took care of him to provide consent to participate in this study. Participants were excluded if they had persistent unconsciousness or were unable to provide ethical consent for their participation and if their caregivers were unable to provide effective information.

## Results

**Figure 1:**
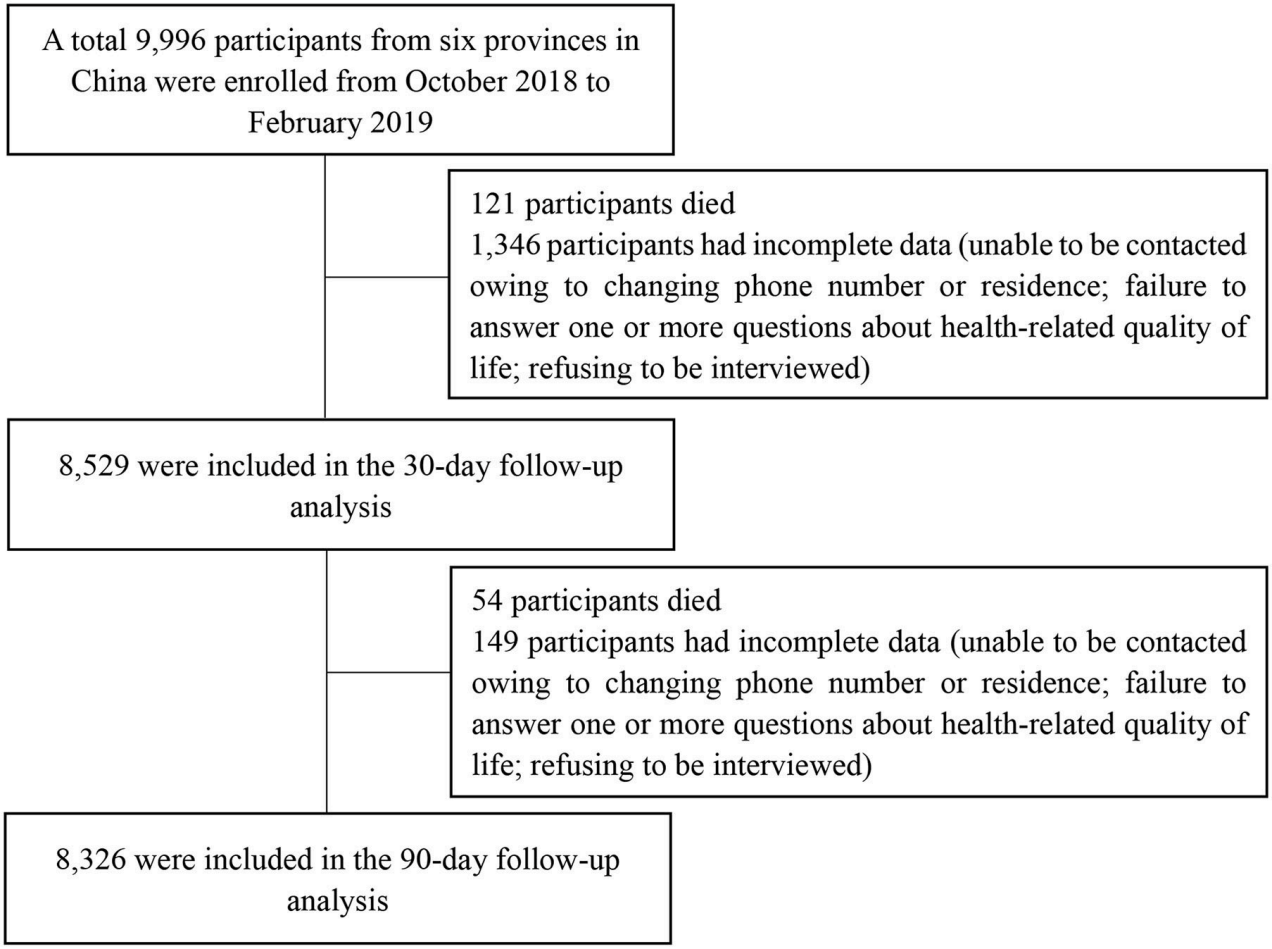
Recruitment and follow-up of participants.

A total of 9,996 elder patients from 314 wards of six hospitals were enrolled in this study. Descriptive statistics and the frequencies of demographics are shown in Table 1. The mean age of this population was 72.47 ± 5.77 years. The average length of stay in the hospital was 9.79 ± 7.56 days. The study flowchart is presented in Figure 1. At the 30-day follow-up, 8,529 (85.3%) participants remained in the study, 121 (1.2%) participants died, and 1,346 (13.5%) had incomplete data (changing phone number or residence permanently, failure to respond to one or more questions about HRQoL, and refusal to be interviewed). At the 90-day follow-up, 8,326 (83.3%) completed the study, 54 (0.5%) participants died, and 149 (1.5%) had incomplete data. At the 30- and 90-day follow-up, the baseline characteristics of those who dropped out of the study (age, sex, frailty, depression, current residence, and nutritional status) did not differ significantly from those who completed it.

**Table 1:** Demographic characteristics of the participants (*N* = 9,996).

**Variables**	**Values**
**Department (*****n*****, 100%)**	
Surgical	3,296 (32.97)
Medicine	4,694 (46.96)
Neurology	970 (9.70)
Orthopedics	719 (7.19)
ICU	317 (3.17)
**Province or municipality/city (*****n*****, 100%)**	
Sichuan Province	1,808 (18.09)
Heilongjiang Province	1,742 (17.43)
Hubei Province	1,824 (18.25)
Beijing municipality/city	1,401 (14.02)
Qinghai Province	1,417 (14.18)
Zhejiang Province	1,804 (18.05)
Length of stay	9.79 ± 7.56
Age	72.47 ± 5.77

*ICU, intensive care unit*.

The general characteristics and nutritional status of the participants completing the study at 30-day follow-up are shown in Table 2. A total 56.7% of the participants were aged 70 years and 57.7% were men; 94.1% of the participants were of Han ethnicity. A total 66.1 and 76.2% of the participants were non-smokers and non-drinkers, respectively. A total 14.3% of the participants had a falling accident in the past 12 months, 42.3% had sleeping difficulties, 12.8% had urinary dysfunction, and 11.3% had defecation dysfunction. The prevalence rates of frailty and depression were 16.4 and 15.4%, respectively. The proportion of participants living in the community was 96.0%. According to the MNA-SF scores, 9.7% of the participants were found to be malnourished; only 6.6% were emaciated, according to the BMI values.

**Table 2:** General characteristics of the participants at the 30-day follow-up (*N* = 8,529).

**Features**			***N***	**%**
Sociodemographic characteristics	Age group (years)	65–69	3,691	43.3
		70–74	2,352	27.6
		75–79	1,490	17.5
		80–84	740	8.7
		85 and above	256	3.00
	Sex	Male	4,922	57.7
		Female	3,607	42.3
	Education	University	1,277	15.0
		Middle school	3,475	40.7
		Primary school	2,423	28.4
		Illiterate	1,354	15.9
	Marital status	Divorced or widowed	930	10.9
		Married	7,599	89.1
	Ethnicity	Han	8,028	94.1
		Others	501	5.9
	Smoking	Non-smoker	5,638	66.1
		Current smoker	943	11.1
		Former smoker	1,948	22.8
	Alcohol drinking	Non-drinker	6,498	76.2
		Current drinker	997	11.7
		Former drinker	1,034	12.1
	Falling accidents in the past 12 months	Yes	1,219	14.3
		No	7,310	85.7
	Sleeping	Normal	4,925	57.7
		Dysfunction	3,604	42.3
	Urinary function	Normal	7,435	87.2
		Dysfunction	1,094	12.8
	Defecation function	Normal	7,569	88.7
		Dysfunction	960	11.3
	Frail	Yes	1,395	16.4
		No	7,134	83.6
	Depression	Yes	1,309	15.4
		No	7,220	84.7
	Current residence	Community	8,187	96.0
		Hospital or nursing home	342	4.0
Nutritional status	MNA-SF	Malnourished (0–7)	823	9.7
		Malnutrition risk (8–11)	2,940	34.5
		Normal (12–14)	4,766	55.9
	BMI (kg/m^2^)	Emaciation (<18.5)	562	6.6
		Normal (18.5–23.9)	4,142	48.6
		Overweight (24–27.9)	2,953	34.6
		Obesity (≥28)	872	10.2

*MNA-SF, Mini Nutritional Assessment—Short Form; BMI, body mass index*.

The descriptive characteristics and nutritional status of the participants completing the study at the 90-day follow-up are presented in Table 3. A total 56.9 and 57.5% of the participants were aged 70 years and are males, respectively. In total, 94.0% of the participants were of Han ethnicity; 66.1 and 76.1% were non-smokers and non-drinkers, respectively. Of the participants, 14.0% had a falling accident in the past 12 months. The proportion of participants with sleeping difficulties was 42.2%, those with urinary dysfunction was 12.9%, and the proportion with defecation dysfunction was 11.1%. The prevalence rates of frailty and depression were 15.9 and 15.1%, respectively. The percentage of participants living in the community was 98.4%. In total, 9.1% of the participants were found to be malnourished, according to the MNA-SF scores; only 6.4% were emaciated, according to the BMI values.

**Table 3:** General characteristics of the participants at the 90-day follow-up (*N* = 8,326).

**Features**			***N***	**%**
Sociodemographic characteristics	Age group (years)	65–69	3,589	43.1
		70–74	2,333	28.0
		75–79	1,453	17.5
		80–84	703	8.4
		85 and above	248	3.0
	Sex	Male	4,786	57.5
		Female	3,540	42.5
	Education	University	1,221	14.7
		Middle school	3,393	40.8
		Primary school	2,400	28.8
		Illiterate	1,312	15.8
	Marital status	Divorced or widowed	933	11.2
		Married	7,393	88.8
	Ethnicity	Han	7,825	94.0
		Others	501	6.0
	Smoking	Non-smoker	5,499	66.1
		Current smoker	929	11.2
		Former smoker	1,898	22.8
	Alcohol drinking	Non-drinker	6,338	76.1
		Current drinker	986	11.8
		Former drinker	1,002	12.0
	Falling accidents in the past 12 months	Yes No	1,168 7,158	14.0 86.0
	Sleeping	Normal	4,810	57.8
		Dysfunction	3,516	42.2
	Urinary function	Normal	7,251	87.1
		Dysfunction	1,075	12.9
	Defecation function	Normal	7,406	89.0
		Dysfunction	920	11.1
	Frail	Yes	1,322	15.9
		No	7,004	84.1
	Depression	Yes	1,254	15.1
		No	7,072	84.9
	Current residence	Community	8,193	98.4
		Hospital or nursing home	133	1.6
Nutritional status	MNA-SF	Malnourished (0–7)	757	9.1
		Malnutrition risk (8–11)	2,831	34.0
		Normal (12–14)	4,738	56.9
	BMI (kg/m^2^)	Emaciation (<18.5)	534	6.4
		Normal (18.5–23.9)	4,037	48.5
		Overweight (24–27.9)	2,909	34.9
		Obesity (≥28)	846	10.2

*MNA-SF, Mini Nutritional Assessment—Short Form; BMI, body mass index*.

At the 30-day follow-up, male sex (regression coefficient, 1.351; 95% CI, 0.567–2.135), normal sleeping (1.335; 0.685–1.985), normal urinary function (1.104; 0.133–2.075), normal defecation function (3.598; 2.575–4.622), and a higher MNA-SF score (0.660; 0.499–0.821) were associated with higher EQ5DVAS scores in the multiple linear regression model. In contrast, age (−0.156; −0.213 to −0.099), former smoker (−1.152; −2.055 to −0.249), falling accidents in the past 12 months (−1.005; −1.906 to −0.104), frailty (−4.587; −5.515 to −3.658), depression (−3.455; −4.429 to −2.481), and living in a hospital or nursing home setting (−13.163; −14.753 to −11.572) were associated with lower EQ5DVAS scores. Contrary to the results of the univariate analysis, the association with BMI values was not significant in the multivariate model (Table 4).

**Table 4:** Variables that can affect the EQ5DVAS score from the regression model (30-day follow-up).

**Characteristics**	**Univariate**		**Multivariate**	
	**Regression coefficient**	**95% CI**	**Regression coefficient**	**95% CI**
Age	−0.243	−0.300 to −0.185	−0.156	−0.213 to −0.099
**Sex**				
Male	1.145	0.478–1.811	1.351	0.567–2.135
Female (ref.)	–	–	–	–
**Education**				
University	0.698	−0.489 to 1.886	−0.727	−1.892 to 0.437
Middle school	0.880	−0.095 to 1.855	−0.621	−1.578 to 0.335
Primary school	0.607	−0.426 to 1.639	−0.472	−1.469 to 0.525
Illiterate (ref.)	–	–	–	–
**Marital status**				
Married	1.213	0.155–2.270	−0.327	−1.363 to 0.709
Divorced or widowed (ref.)	–	–	–	–
**Ethnicity**				
Han	1.074	−0.327 to 2.476	0.054	−1.305 to 1.413
Others (ref.)	–	–	–	–
Smoking				
Current smoker	0.817	−0.254 to 1.887	−0.522	−1.641 to 0.597
Former smoker	−0.434	−1.234 to 0.366	−1.152	−2.055 to −0.249
Non-smoker (ref.)	–	–	–	–
**Alcohol drinking**				
Current drinker	1.736	0.702–2.771	0.205	−0.866 to 1.275
Former drinker	−0.273	−1.292 to 0.745	−0.201	−1.284 to 0.882
Non-drinker (ref.)	–	–	–	–
**Falling accidents in the past 12 months**				
Yes	−2.533	−3.474 to −1.593	−1.005	−1.906 to −0.104
No (ref.)	–	–	–	–
**Sleeping**				
Normal	3.155	2.491–3.819	1.335	0.685–1.985
Dysfunction (ref.)	–	–	–	–
**Urinary function**				
Normal	3.402	2.419, 4.385	1.104	0.133, 2.075
Dysfunction (ref.)	–	–	–	–
**Defecation function**				
Normal	6.466	5.432, 7.500	3.598	2.575, 4.622
Dysfunction (ref.)	–	–	–	–
**Frail**				
Yes	−8.584	−9.456 to −7.712	−4.587	−5.515 to −3.658
No (ref.)	–	–	–	–
**Depression**				
Yes	−7.558	−8.458 to −6.657	−3.455	−4.429 to −2.481
No (ref.)	–	–	–	–
**Current residence**				
Hospital or nursing home	−14.502	−16.154 to −12.850	−13.163	−14.753 to −11.572
Community (ref.)	–	–	–	–
MNA-SF	1.300	1.174–1.42	0.660	0.499–0.821
BMI	0.363	0.269, 0.457	−0.001	−0.104 to 0.102

*All models were adjusted for age, sex, education, marital status, ethnicity, smoking, alcohol drinking, falling accidents in the past 12 months, sleeping, urinary function, defecation function, frailty, depression, and current residence*.

*MNA-SF, Mini Nutritional Assessment—Short Form; EQ5DVAS, EuroQoL Five-Dimension Visual Analog Scale; BMI, body mass index; CI, confidence interval*.

At the 90-day follow-up, current drinker (regression coefficient, 1.623; 95% CI, 0.407–2.838), normal sleeping (1.145; 0.403–1.888), normal urinary function (2.147; 1.045–3.249), normal defecation function (3.581; 2.404–4.758), and a higher MNA-SF score (0.622; 0.434–0.809) were associated with higher EQ5DVAS scores in the multiple linear regression model. Age (−0.144; −0.210 to −0.078), Han ethnicity (−1.569; −3.104 to −0.035), former smoker (−1.291; −2.325 to −0.256), falling accidents in the past 12 months (−1.270; −2.306 to −0.233), frailty (−4.187; −5.264 to −3.111), depression (−3.448; −4.571 to −2.326), and living in hospital or a nursing home (−16.029; −18.874 to −13.185) were associated with lower EQ5DVAS scores. Contrary to the results of the univariate analysis, the association with BMI values was not significant in the multivariate model (Table 5).

**Table 5:** Variables that can affect the EQ5DVAS score from the regression model (90-day follow-up).

**Characteristics**	**Univariate**		**Multivariate**	
	**Regression coefficient**	**95% CI**	**Regression coefficient**	**95% CI**
Age	−0.239	−0.304 to −0.174	−0.144	−0.210 to −0.078
**Sex**				
Male	0.009	−0.758 to 0.739	−0.335	−1.242 to 0.572
Female (ref.)	–	–	–	–
**Education**				
University	1.848	0.506–3.189	1.122	−0.218 to 2.463
Middle school	0.052	−1.045 to 1.149	−0.773	−1.870 to 0.323
Primary school	0.456	−0.723 to 1.594	−0.118	−1.257 to 1.021
Illiterate (ref.)	–	–	–	–
**Marital status**				
Married	1.540	0.367–2.712	0.364	−0.811 to 1.538
Divorced or widowed (ref.)	–	–	–	–
**Ethnicity**				
Han	−0.286	−1.841 to 1.270	−1.569	−3.104 to −0.035
Others (ref.)	–	–	–	–
**Smoking**				
Current smoker	0.687	−0.509 to 1.884	−0.178	−1.453 to 1.098
Former smoker	−1.059	−1.957 to −0.160	−1.291	−2.325 to −0.256
Non-smoker (ref.)	–	–	–	–
**Alcohol drinking**				
Current drinker	2.395	1.240, 3.549	1.623	0.407, 2.838
Former drinker	−0.537	−1.684 to 0.609	0.386	−0.853 to 1.625
Non-drinker (ref.)	–	–	–	–
**Falling accidents in the past 12 months**				
Yes	−2.604	−3.668 to −1.540	−1.270	−2.306 to −0.233
No (ref.)	–	–	–	–
**Sleeping**				
Normal	2.928	2.182, 3.675	1.145	0.403, 1.888
Dysfunction (ref.)	–	–	–	–
**Urinary function**				
Normal	4.302	3.202–5.401	2.147	1.045–3.249
Dysfunction (ref.)	–	–	–	–
**Defecation function**				
Normal	6.413	5.241–7.558	3.581	2.404–4.758
Dysfunction (ref.)	–	–	–	–
**Frail**				
Yes	−8.144	−9.141 to −7.147	−4.187	−5.264 to −3.111
No (ref.)	–	–	–	–
**Depression**				
Yes	−7.448	−8.471 to −6.426	−3.448	−4.571 to −2.326
No (ref.)	–	–	–	–
**Current residence**				
Hospital or nursing home	−18.510	−21.434 to −15.587	−16.029	−18.874 to −13.185
Community (ref.)	–	–	–	–
MNA-SF	1.267	1.121–1.412	0.622	0.434–0.809
BMI	0.363	0.256–0.470	0.017	−0.102 to 0.136

*All models were adjusted for age, sex, education, marital status, ethnicity, smoking, alcohol drinking, falling accidents in the past 12 months, sleeping, urinary function, defecation function, frailty, depression, and current residence*.

*MNA-SF, Mini Nutritional Assessment—Short Form; EQ5DVAS, EuroQoL Five-Dimension Visual Analog Scale; BMI, body mass index; CI, confidence interval*.

## Discussion

To our knowledge, this is the first study to examine the associations between nutritional status, sociodemographic characteristics, and health-related variables and HRQoL among a nationally representative sample of Chinese hospitalized elderly population in tertiary hospitals. The main finding was that higher MNA-SF scores were related to higher HRQoL scores in elderly patients, both at the 30- and 90-day follow-up. However, we found no significant associations between the BMI values and HRQoL. Sociodemographic characteristics (such as age, smoking, and current residence), physical health variables (frailty, urinary function, defecation function, sleeping condition, and falling accidents in the past 12 months), and mental health variables (depression) were the main factors influencing HRQoL in this group.

The MNA-SF scores showed significant associations with the HRQoL scores, suggesting that nutritional status may have an important influence on the HRQoL among the elderly. This finding is consistent with previous studies reporting that the HRQoL scores decrease with the decline of the MNA-SF scores (6, 8, 13). Poor nutrition leads to ill health and ill health to poor nutrition, so identifying priorities for management is a key issue as well as a challenge. One study from Turkey reported that a 12-week intervention with oral nutritional supplementation plus physical exercise improved the nutritional status and HRQoL in older adults (13). Therefore, developing targeted nutritional supplementation plans to help older people to improve their HRQoL is fundamental to the management of healthy aging among older individuals in China. In addition, despite growing evidence that nutritional support improves HRQoL in older people, more data are urgently needed regarding the effects of nutritional support on HRQoL in the elderly.

In this study, MNA-SF and BMI were assessed at the time of admission to the hospital, and HRQoL was recorded at 30 and 90 days after admission via follow-up regardless of whether the participant is hospitalized or discharged. Generally, MNA-SF may reflect the nutritional status in the past 3 months; thus, the MNA-SF is more representative of the long-term nutritional status of participants than does BMI. Accordingly, the potential association between MNA-SF and HRQoL is more convincing in reflecting the relationship between nutritional status and clinical outcomes. BMI is prone to changes owing to weight gain or loss during hospitalization, which could explain the statistically non-significant results between BMI and HRQoL (8). Still, we believe that controlling BMI is essential to improving the clinical outcomes, as many researchers have shown that BMI is closely related to patients' inflammation and oxidation levels. Further research is required to confirm the relationships between nutritional status and clinical outcomes in this regard.

Similar to the present results, some studies have shown that HRQoL indicators decrease with aging in older adults (25, 37–39). In addition, our results suggest that the HRQoL scores among the participants in the hospital or nursing home settings were lower compared to the participants living in the community. This is perhaps because the community-dwelling elderly can receive support from family, friends, and neighbors and assistance with activities of daily living, such as the sourcing and preparation of meals and help with eating, when necessary (40), which means that such individuals have a better quality of life. Therefore, investing in adequate geriatric care sources, developing a home care-dominated system, supported by community care, and supplemented with institutional care aimed at helping the elderly to self-manage their daily activities and improving their HRQoL are warranted.

Apart from age and residence, former smokers had lower HRQoL scores than non-smokers. Previous studies have also demonstrated a negative relationship between smoking and HRQoL and that smoking cessation significantly improves HRQoL (41).

With regard to the health-related variables, our results suggest that physical and mental health variables including frailty, depression, and falling accidents in the past 12 months can bring about a series of negative effects on HRQoL; other research support these findings. In a study conducted among older adults, frail participants had leaner body mass, lower HRQoL scores, greater depression, and greater vulnerability to falls than the non-frail participants (9). Schoene et al. (42) demonstrated that a history of falling accidents might greatly affect HRQoL in older people. Other studies have reported that depression has a major influence on HRQoL, which can affect general appetite, and the HRQoL scores decrease with decreased general appetite (8, 39, 43). Besides, HRQoL was positively correlated with urinary function, defecation function, and sleeping condition in the present study. Regarding these factors, early assessment, identification, and prevention are important. More advanced risk factor assessment scales, standardized nursing care measures, and nutrition and physical activity interventions may be useful in improving the HRQoL among elderly patients in the future.

Compared with women, the men in our study had a lower risk of poor HRQoL at the 30-day follow-up; in addition, HRQoL was poorer in women than in men, as reported in other studies (44–46). This is perhaps because men generally have better muscle storage than women, and that may affect physical conditions (such as frailty), appetite, and other factors linked to HRQoL (47–49). At the 90-day follow-up, Han ethnicity was associated with lower HRQoL scores than did other ethnicities, which might be owing to differences in the ethnic distribution of our participants. In addition, current drinkers had higher HRQoL scores than non-drinkers; this can be ascribed to the fact that older adults who consume small to moderate amounts of alcohol are more likely to maintain physical functioning than non-drinkers, as moderate alcohol consumption has been associated with a decreased risk of cardiovascular disease (19). However, detailed assessments based on the amount and frequency of alcohol consumption are needed.

This study has some limitations that should be considered when interpreting the results. First, because of convenience sampling, the patients enrolled in our study were selected from tertiary hospitals and from only one hospital in each province or municipality/city, which limited the generalizability of this study. Second, there were a considerable number of potential participants who were unable to be contacted, most of whom were in distant provinces or were migrant workers; this may have led to selection bias. Thirdly, although nutritional status and HRQoL can be assessed using several different assessment scales, our use of a limited number of measurement tools restricted the comparison of our results with those of other studies. Fourth, the participants enrolled in this study were relatively young—nearly half of the participants were 65–69 years old—which limited the generalizability of this study. Fifth, the reason for the admission might be a major factor influencing HRQoL. The target population in this study involved multiple wards or departments; we did not analyze the reason for the admission, nor did we analyze the application of medical and nursing care in older patients. Finally, chronic diseases (such as cancer, diabetes, and cardiovascular diseases) could be associated with poor HRQoL among elderly patients. The participants in this study covered many departments, and we did not analyze the impact of chronic diseases in this paper. Prospective studies with more sophisticated evaluations are required in the future.

## Conclusion

This study suggests that higher MNA-SF scores were associated with higher HRQoL scores in Chinese elderly patients, whereas there were no significant associations between BMI and HRQoL. Sociodemographic characteristics and physical and mental health variables were the main factors influencing HRQoL in this group. Investing in adequate geriatric care sources, developing a home care-dominated system, supported by community care, and supplemented with institutional care aimed at improving HRQoL are warranted. In light of the factors associated with HRQoL, attention should be paid to risk assessment, preventive measures, nutrition, and physical activity intervention. These findings could have major importance in the planning of “active aging” policies and programs.

## Data Availability Statement

The datasets used and analyzed during the current study are available from the corresponding author on reasonable request.

## Ethics Statement

The studies involving human participants were reviewed and approved by the ethics committee of Peking Union Medical College Hospital (S-K540). The patients/participants provided their written informed consent to participate in this study.

## Author Contributions

XWu conceived and designed this study. HL prepared and edited the manuscript and drafted the tables. JJia and TX performed the statistical analyses and reviewed the manuscript. CZ, MZ, XWe, JJin, HW, DL, and SZ recruited participants, collected data, and edited the manuscript. All authors read and approved the final manuscript.

## Conflict of Interest

The authors declare that the research was conducted in the absence of any commercial or financial relationships that could be construed as a potential conflict of interest.

## Abbreviations

MNA-SF: Mini Nutritional Assessment—Short Form
BMI: body mass index
ICU: intensive care unit
CI: confidence interval
EQ5DVAS: EuroQoL five-dimension visual analog scale
HRQoL: health-related quality of life.
